# Successful Neurological Recovery with Multimodality Therapy without Surgery for Spinal Metastases from Advanced Gastric Cancer

**DOI:** 10.1155/2020/4753027

**Published:** 2020-02-06

**Authors:** Hideyuki Kinoshita, Hiroto Kamoda, Takeshi Ishii, Yoko Hagiwara, Toshinori Tsukanishi, Yusuke Amanuma, Rino Nankinzan, Sumihisa Orita, Kazuhide Inage, Naoya Hirosawa, Seiji Ohtori, Tsukasa Yonemoto

**Affiliations:** ^1^Department of Orthopedic Surgery, Chiba Cancer Center, 666-2 Nitonacho, Chuo-ku, Chiba 260-8717, Japan; ^2^Department of Gastroenterology, Chiba Cancer Center, 666-2 Nitonacho, Chuo-ku, Chiba 260-8717, Japan; ^3^Department of Endoscopy, Chiba Cancer Center, 666-2 Nitonacho, Chuo-ku, Chiba 260-8717, Japan; ^4^Department of Orthopaedic Surgery, Graduate School of Medicine, Chiba University, 1-8-1 Inohana, Chuo-ku, Chiba 260-8670, Japan

## Abstract

Advanced gastric cancer with bone metastasis has a very poor prognosis with short median survival. To the best of our knowledge, no reports in literature have described extensive recovery of paralysis with multimodality treatment without surgery in these cases. This report describes the case of a 52-year-old severely paralyzed female patient with spinal metastasis from advanced gastric cancer. She was inoperable, owing to a large thrombus in the inferior vena cava; alternative multimodality treatments, including chemotherapy and radiotherapy, were administered. The paralysis and the bladder and rectal dysfunction improved considerably. In addition, the performance status (PS) and Frankel grade also improved dramatically, from 4 to 1 and grade B to D, respectively. At 1 year after initiation of treatment, she is ambulatory. Patients with poor PS are often offered palliative therapy. However, this case demonstrates that poor PS solely due to paralysis from spinal metastasis may necessitate multimodality treatment instead of palliative care.

## 1. Introduction

Metastases to the bone are common and are indicative of short survival in patients with cancer. The most common sites involved are the spine, pelvis, ribs, skull, and proximal femur. Spinal metastases occur in approximately 40% of cancers with occult lesions [1]. Almost 20% of patients diagnosed with spinal metastases subsequently develop symptomatic spinal cord compression [2], requiring multimodality treatment, including surgery, chemotherapy, and radiotherapy. Gastric cancer continues to be one of the most common and deadly cancers worldwide. The median survival in advanced gastric cancer with bone metastases is considerably short; decision-making for management is therefore difficult. Extensive neurological recovery of paralysis is very rare following multimodality treatment for metastatic spinal compression.

This report describes a rare case of neurologic deficit due to spinal metastasis from advanced gastric cancer that improved with multimodality treatment without surgery.

## 2. Case Presentation

**Figure 1:**
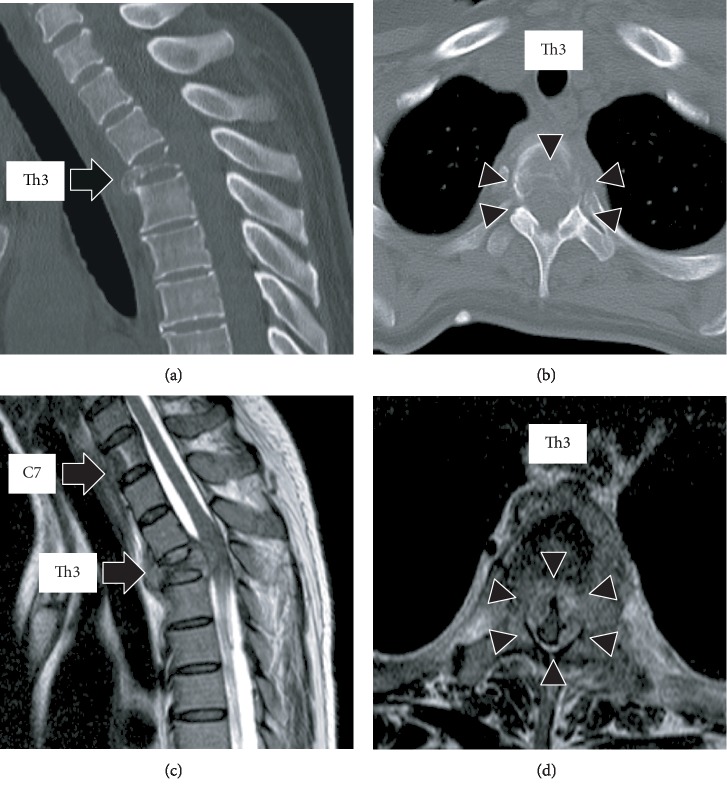
(a, b) Sagittal and axial views of the thoracic spine on computed tomography (CT) before treatment showing a collapsed Th3 vertebral body (arrow) due to the lytic lesion (arrowhead), causing kyphosis. (c, d) Sagittal and axial views of the thoracic spine on magnetic resonance imaging (MRI) showing C7 and Th3 vertebral bodies (arrow) infiltrated by the tumor. At the Th3 level, the tumor extends into the posterior vertebral body and bilateral pedicles, resulting in severe compression of the spinal cord (arrowhead).

**Figure 2:**
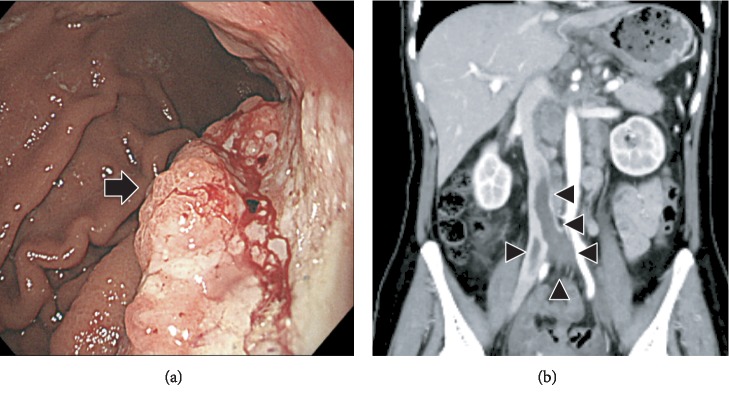
(a) Gastroscopy showing elevated lesions with bleeding in the body of the stomach (arrow). (b) Whole-body CT showing a large thrombus extending from the bilateral common iliac veins to the inferior vena cava (arrowhead).

**Figure 3:**
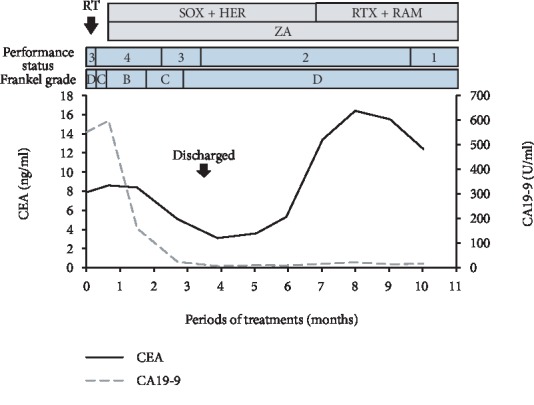
Changes in carcinoembryonic antigen (CEA) levels, carbohydrate antigen 19-9 (CA19-9) performance status (PS), and Frankel grade during treatment with the flow chart of radiotherapy and chemotherapy. First-line and second-line chemotherapy included 9 courses of SOX-HER with ZA and 4 courses of PTX-RAM with ZA, respectively. RT: radiation therapy (single 8 Gy fraction); SOX: tegafur/gimeracil/oteracil (40 mg/m^2^/day for 2 weeks, followed by 1 week of no medication)+oxaliplatin (130 mg/m^2^ every 3 weeks); HER: trastuzumab (6 mg/kg every 3 weeks); PTX: paclitaxel (80 mg/m^2^ every 2 weeks); RAM: ramucirumab (8 mg/kg every 2 weeks); ZA: zoledronic acid (4 mg every 4 weeks).

**Figure 4:**
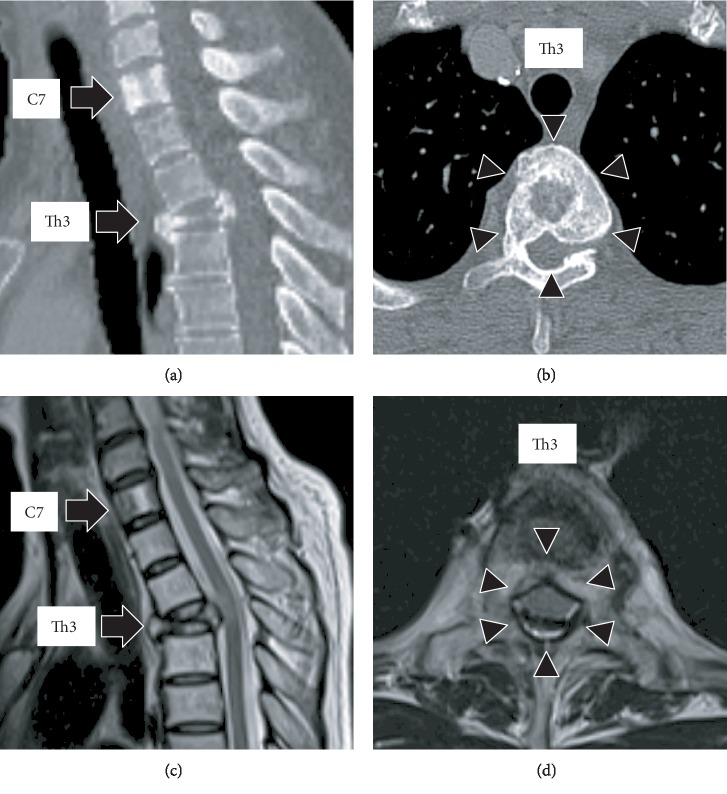
(a, b) Sagittal and axial views of the thoracic spine on computed tomography (CT) after 1 year of multimodality treatment showing osteosclerosis (arrowhead) of the C7 and Th3 vertebral bodies (arrow). (c, d) Sagittal and axial views of the magnetic resonance imaging (MRI) after 1 year of multimodality treatment showing tumor shrinkage in the C7 and Th3 vertebral bodies (arrow), enlarging the spinal canal (arrowhead).

A 52-year-old female, with no history of illness, medication, fever, trauma, weight loss, or previous infection, consulted local clinics for severe back pain, which had lasted for 2 months. She was advised rest at home. After 2 months, she developed paralysis in the lower limbs with continuing severe back pain, for which she was transported to our hospital. On examination, she had bilateral lower limb proximal muscle weakness (manual muscle testing level 3 and Frankel grade D) with a PS score of 3. Computed tomography (CT) revealed collapse of the Th3 vertebral body due to a lytic lesion, which caused kyphosis (Figures 1(a) and 1(b)). On magnetic resonance imaging (MRI), the C7 and Th3 vertebral bodies were infiltrated by a tumor (Figure 1(c)). At the Th3 level, the tumor extended into the posterior vertebral body and bilateral pedicles, resulting in severe compression of the spinal cord (Figure 1(d)). Blood tests revealed severe anemia with a hemoglobin (Hb) level of 5.8 g/dl; the serum carcinoembryonic antigen (CEA) and carbohydrate antigen 19-9 (CA19-9) were high at 7.9 ng/ml and 551 U/ml, respectively, arousing suspicion of gastric cancer. Gastroscopy revealed elevated lesions with bleeding in the body of the stomach; histopathological examination of the biopsy specimen revealed adenocarcinoma; the HER2 score was 3+ (Figure 2(a)). The whole-body CT showed a large thrombus extending from the bilateral common iliac veins to the inferior vena cava, probably caused by immobility; neck and axillary lymph node enlargement was also found (Figure 2(b)). Edoxaban (30 mg/day) was administered to dissolve the large thrombus in the inferior vena cava. The neurologic symptoms were gradually exacerbated with emergence of bladder and rectal dysfunction and the Frankel grade was C. Posterior decompression of the thoracic spine was planned urgently. However, general anesthesia and surgery were not feasible due to the large thrombus in the inferior vena cava. The power of the proximal leg muscles gradually deteriorated and the Frankel grade and performance status score worsened to B and 4, respectively. Palliative radiotherapy (RT) was delivered urgently to the Th3 vertebra (single 8 Gy fraction). Chemotherapy was initiated after consultation with the digestive department. She was prescribed tegafur/gimeracil/oteracil, oxaliplatin, and trastuzumab as first-line therapy based on evidence from a phase 3 clinical study [3]. A bisphosphonate, namely, zoledronic acid (4 mg every 4 weeks), was also instituted to control bone metastases. The neurological symptoms began to improve dramatically from 1 month after initiating chemotherapy (Figure 3). Remarkably, the large thrombus in the inferior vena cava disappeared after continuing edoxaban for 3 months. Three months since the initiation of chemotherapy, the Hb and tumor marker values improved to normal and the patient was able to stand and walk with a pickup walker and was discharged home. Since the CEA was elevated and the para-aortic lymph nodes were enlarged 6 months after tegafur/gimeracil/oteracil, oxaliplatin, and trastuzumab were started, paclitaxel and ramucirumab were prescribed as a second-line therapy (Figure 3). At 1 year from the initiation of multimodality treatment, the follow-up CT demonstrated osteosclerosis in the affected vertebra (Figures 4(a) and 4(b)). Furthermore, MRI demonstrated that the tumor had shrunk with relief of spinal compression (Figure 4(c) and 4(d)). The patient is now able to walk independently for a distance of 5 m and the bladder and rectal dysfunction has resolved. The course of the disease is good with no evidence of tumor progression.

## 3. Discussion

The bone is a common site of metastasis for carcinomas of the prostate, breast, lung, kidney, bladder, and thyroid. Owing to a worldwide increase in the incidence of cancer and a longer life expectancy of patients with metastatic cancer, the incidence of symptomatic vertebral metastases has risen [4]. Although surgical treatment of spinal metastases remains controversial, Tangpatanasombat et al. reported that patients with spinal metastases benefit from palliative surgery, with significant pain relief and neurological recovery [5]. However, depending on the primary site and histological types, the prognosis is highly variable [6]. Although spinal metastases from lymphoma are often successfully managed by surgery or other modalities, the prognosis in case of other cancers, including those of the bladder and stomach, are often poor [7]. Gastric cancer rarely disseminates to the bones, the incidence being only 0.9-3.8% [8]. Riihimäki et al. reported that in gastric cancer, bone metastasis was associated with the shortest median survival compared to metastases in other sites, including the thorax, liver, or other regions of the abdomen [9]. Although Zhong et al. reported that surgery is an efficient option in treating gastric cancer spinal metastasis [10], owing to the poor survival in these cases, surgery tends to rarely be performed to relieve paralysis from spinal compression in many institutions. To the best of our knowledge, successful outcomes are very rare following medical or surgical treatment for paralysis due to spinal metastases from gastric cancer.

The revised Tokuhashi score for predicting prognosis in spinal metastases (range: 0-15 points) classified postoperative survival into 3 time periods, namely, mortality within 6 months, 12 months, and more than 12 months [11]. The current case scored 2 points on the revised Tokuhashi score, predicting mortality within 6 months. As per the gastroenterologists expectations, survival in this case would be less than 3 months or less than 1 year in the event of poor and good response to chemotherapy, respectively. In the present case, the tumor responded remarkably to chemotherapy and the patient is well and surviving for about 1 year. In addition, the PS and Frankel grade have also improved dramatically, from 4 to 1 and grade B to D, respectively. Ambulation recovered and she can now walk a distance of 5 m independently. After 1 year, the MRI showed marked tumor shrinkage in the spinal canal.

Gastric cancer remains one of the most common and deadly cancers worldwide, particularly among older males. Based on the GLOBOCAN 2018 data, stomach cancer is the fifth most common neoplasm and the third most deadly, with an estimated 783,000 deaths in 2018 [12]. Patients with progressive gastric cancer experience several symptoms, including anorexia, fatigue, abdominal distension, and dyspnea, which may worsen the general condition of the patient [13]. Cases presenting with bone metastases without any preceding gastrointestinal symptoms make diagnosis particularly difficult [14–16]. In the present case, the patient had no abdominal symptoms despite an advanced gastric cancer. The only sign was anemia without bloody stools; 7 days elapsed before a diagnosis could be made. It is therefore prudent to consider the possibility of gastric cancer in such cases. The median survival in advanced gastric cancer is known to be considerably short, ranging between 6 and 14 months [3, 17]. PS is known to be a strong independent prognostic factor for survival in these patients [18]. Furthermore, Catz et al. reported that in cases of spinal injury, neurologic recovery was negatively associated with the severity of the neurologic deficit [19]. The improvement in the present case was dramatic despite the poor PS and severe paralysis; this is extremely rare.

Currently, the major treatment guidelines for advanced gastric cancers include those from the National Comprehensive Cancer Network (NCCN), European Society of Medical Oncology (ESMO), and the Japanese Gastric Cancer Association (JGCA) [20]. As per these guidelines, HER2 scoring, evaluated by immunostaining and fluorescence in situ hybridization (FISH) is of particular importance in the treatment of unresectable advanced gastric cancer [21]. As per the Japanese guidelines, tegafur/gimeracil/oteracil with either cisplatin or oxaliplatin are mainly used as primary chemotherapy in HER2-negative cases. In HER2-positive cases, trastuzumab is added to this regimen. Cases resistant to first-line chemotherapy require second-line regimens. The Japanese guideline recommends a combination of paclitaxel and ramucirumab based on the results of the RAINBOW phase 3 trial [22]. Since the present patient had a HER2-positive tumor, tegafur/gimeracil/oteracil with oxaliplatin and trastuzumab was administered with gradual improvement of symptoms 1 month after chemotherapy initiation. However, as the para-aortic lymph nodes were enlarged and the CEA was elevated 6 months after the initiation of first-line chemotherapy, the patient was switched over to second-line therapy comprising paclitaxel and ramucirumab, which shrunk the involved lymph nodes. Bisphosphonates play an important role in the treatment of several cancers, particularly of bone metastases, by reducing the risk of fractures, spinal cord compression, and hypercalcemia. Preclinical studies have demonstrated that zoledronic acid may inhibit angiogenesis, invasion, and adhesion of tumor cells [23]. Although many reports describe surgical and endoscopic treatment for gastric cancer, there are few reports on palliative radiation therapy [24]. However, being minimally invasive, radiotherapy is a viable treatment option for palliative treatment in this condition [25]. In the present case, multimodality treatment combined with chemotherapy, a bone-modifying agent, and radiotherapy was successful in achieving a favorable response without surgery.

The present case was inoperable owing to a large thrombus in the inferior vena cava. Several reports confirm the efficacy of edoxaban in treating deep vein thrombosis (DVT) and pulmonary thromboembolism in patients with cancer [26, 27]. Streiff et al. recommended early administration of direct oral anticoagulants (DOACs) such as edoxaban instead of conventional heparin or warfarin for treating DVT and pulmonary embolism [28].

In conclusion, the present report describes a rare case of extensive neurological recovery from compressive myelopathy due to spinal metastasis from advanced gastric cancer, using multimodality treatment without surgery. The poor prognosis of gastric cancer with bone metastases, in conjunction with a poor PS, often prompt treatment with palliative intent. However, in cases where the decline in PS is solely attributable to paralysis, multimodality treatment may be more promising than palliative therapy, which intends to relieve compressive myelopathy from bone metastases.
